# Low Emotional Intelligence: A Precursor of Mental Health Derangements Among Adolescents

**DOI:** 10.7759/cureus.46321

**Published:** 2023-10-01

**Authors:** Priya Y Kulkarni, Gajanan Velhal

**Affiliations:** 1 Community Medicine, Symbiosis Medical College for Women, Pune, IND; 2 Community Medicine, BKL Walawalkar Rural Medical College, Chiplun, IND

**Keywords:** mental health disorders, depression anxiety stress, adolescents, mental health, emotional intelligence

## Abstract

Introduction: Emotional intelligence (EI) is the most researched psychological construct in the 21st century. It predicts success and happiness in life and is suggested as a predictor of mental health (MH). We aimed to assess whether low EI among adolescents acts as a precursor of their MH derangements.

Materials and methods: A cross-sectional study was carried out in Pune Municipal Corporation in 2021 with all due approvals, consent, and assent. EI and MH of adolescents studying in Xth standard in randomly selected 24 out of 440 secondary schools were assessed by Schutte’s Emotional Intelligence Test (SET) and Depression, Anxiety, and Stress Scale 42 (DASS-42) with collection of socio-demographic information. The presence of symptoms of mild to extremely severe depression, anxiety, and stress was considered as MH derangement. All research instruments were translated into the local language, pre-tested, and validated before use. Class teachers were trained for data collection. Data were imported to SPSS version 20 (IBM Corp., Armonk, NY) data editor for further analysis. After enlisting frequencies and proportions, associations and correlations were tested by the chi-squared test and Spearman correlation coefficient, respectively.

Results: A total of 622 participants submitted all research instruments. The mean age was 14.74 (+0.742) years. Boys and girls were 38% and 62%, respectively. The majority were Hindus, belonging to socio-economic classes II and III, residing in urban areas. Symptoms of severe to extremely severe depression and anxiety, but not stress, were associated with low EI (p < 0.0001, 0.001, and 0.229). Also, the EI score had a negative correlation with the depression score (ρ = -0.221, p < 0.0001) and anxiety score (ρ = -0.152, p = 0.001), but not with the stress score.

Conclusion: Low EI can be taken as a precursor of MH derangements, especially in the form of depression and anxiety among school-going adolescents.

Recommendations: Efforts to improve EI among adolescents will help to decrease MH derangements, subsequent MH disorders, and suicidality, with improvement in academic performance.

## Introduction

Mental health (MH) is an essential component of overall health. It is a state of well-being in which an individual realizes his or her own abilities, can cope with normal stresses of life, and can work productively and fruitfully [1].

Recently, there has been a significant increase in trends for self-reported family and academic distress, generalized and social anxiety, and depression [2].

The current state of adolescents’ MH is labeled as a “crisis” as MH derangements have increased in this age group dramatically in the present era. This age group has been reported to have the highest rate of MH disorders at approximately 20%. Every sixth individual in the world and every fifth individual in India is an adolescent and in 2019, it was estimated that one in seven adolescents experiences some form of MH disorder. In India, almost one in 20 people over 18 years suffer the consequences of depression once in a lifetime with roots being in adolescence [3]. The youth population of the 15-19 years age group in India has the highest rate of suicide in the world [4] and is the fourth leading cause of death among 15-19-year-olds in India [4].

The majority of adult MH disorders (MHDs) have their first onset in childhood and adolescence. Hence, it is of utmost importance to detect the emergence of MH issues among adolescents at the initial stage of its development for timely management to prevent its devastating outcomes [5].

One proposed concept to prevent MHDs is that of emotional intelligence (EI) [6]. It is the ability to carry out accurate reasoning about emotions. Higher EI predicts sound MH and more happiness and quality of life [7]. Logical-mathematical intelligence quotient (IQ) helps us in calculations to resolve problems or to process information. Though people with high IQs have better mental abilities, IQ cannot be improved after a certain age [8]. Secondly, IQ has low predictability in real-life situations and workplaces. It re-emphasized the role of sound EI, which helps us to adjust ourselves and others’ emotions. Researchers have shown that the majority of success depends on EI. Higher EI appears to be a good predictor of lower MHDs among adults [9].

The point of significance of EI is that it can be modified at any time all over the life and so the general and mental health. Westerns have researched EI to be a protective factor against MHDs. Among adolescents, it increases success, happiness, and resilience and decreases school burnout [10].

Hence, low EI may make individuals prone to develop MHDs. In such individuals, if EI is improved, their quality of life can be improved due to the experience of more happiness and success.

Such relation between EI and MH needs to be assessed qualitatively and quantitatively among Indian adolescents who constitute nearly 20% of the Indian population and recently have shown an increased prevalence of MHDs and suicidality [3]. It is of utmost importance to identify MH derangements and their precursors existing among adolescents as early as possible to halt their progress to disabling MHDs and consequent adverse outcomes.

Hence, in this study, we aimed to assess whether low EI can be taken as a precursor of MH derangements among adolescents in India.

## Materials and methods

It was a cross-sectional study conducted in the first half of 2021 among adolescents studying in 10th standard in secondary schools located in one of the municipal corporations in India. The study was preceded by approval from the Institutional Ethics Committee (IEC) of Maharashtra Institute of Medical Education and Research (MIMER), Pune (approval number: IEC/540), and permission of the education officer, Pune Municipal Corporation. As study participants were minor, parental consent was taken followed by assent of study participants.

The sample size was calculated as 597, assuming the anticipated prevalence of MH derangements among adolescents as 54% from previous studies in the same study area and 4% of allowable error [11]. It was rounded to 622. A total of 24 secondary schools following the state board syllabus pattern were selected in the municipal corporation under study. As per the COVID-19 restrictions, school education was being continued through online and web-based modes. The same was utilized for data collection and for obtaining consent from parents and the assent of study participants. Eligible participants who did not have access to online education were excluded from the study.

Schutte's Emotional Intelligence Test (SET) was used as a measure of general EI of study subjects [12]. It is a 33-item questionnaire to be self-reported on a Likert scale ranging from 1 (strongly agree) to 5 (strongly disagree). Its items relate to six aspects of EIs: (1) appraisal of others’ emotions; (2) appraisal of own emotions; (3) regulation of emotion; (4) social skills; (5) utilization of emotion; (6) optimism [13]. It has internal consistency reliability as r = 0.87 to 0.90 and two-week test-retest reliability (r = 0.78; r - Pearson’s correlation coefficient) [13].

MH of study participants was assessed by the presence of symptoms of depression, anxiety, and stress (DAS) and their severity. The presence of moderate to extremely severe symptoms of any of DAS was considered MH derangement. It was assessed using the Depression, Anxiety, and Stress Scale 42 (DASS-42), which is a valid psychometric tool. Its Cronbach’s alpha coefficient for DAS was 0.85, 0.81, and 0.8 in 2016 to 0.94, 0.88, and 0.93 in 2003, respectively [14]. It has a total of 42 statements, 14 statements for each to assess DAS. It is to be self-reported on a Likert scale ranging from 0 (never) to 3 (many times) based on how much these statements applied to the recipient in the past week [15].

Socio-demographic information of the study participants was collected by semi-structured, self-administered questionnaire in the local language. Socioeconomic status (SES) was assessed by the modified Kuppuswamy's socioeconomic scale based on the 2021 Consumer Price Index [16].

All research instruments were converted into the local language, pre-tested, and validated before data collection. Study participants were explained how to answer SET and DASS-42 on a Likert scale. Approximately 10 minutes were required to complete one questionnaire. Data were entered in MS Excel software (Microsoft Corporation, Redmond, WA). DASS-42 and SET responses were summated and categorized as per conventions laid by their inventors (Table 1). DAS categories were re-divided as "no to mild" and "moderate to extremely severe" as per chances of progression to frank MHDs in the future.

**Table 1:** Categorization of DAS and EI scores DAS: depression, anxiety, and stress; EI: emotional intelligence.

DAS/EI	No evidence	Mild	Moderate	Severe	Extremely severe
Depression	0–9	10–13	14–20	21–27	≥28
Anxiety	0–7	8–9	10–14	15–19	≥20
Stress	0–14	15–18	19–25	26–33	≥34
EI	Low		Normal	High	
	<110		111-137	>137	

Data were imported to SPSS version 20 (IBM Corp., Armonk, NY) data editor for further analysis. Frequencies and proportions of sociodemographic variables under study and categories of DAS and EI were enlisted. Associations of DAS and EI categories were tested by the chi-squared test, and the correlation between DAS and EI scores was tested by the Spearman correlation coefficient.

## Results

Baseline characteristics

A total of 622 participants completed all three research instruments. The mean age of the study participants was 14.74 (+0.742) years; 38% were boys while 62% were girls. The majority of them were Hindu by religion. About, 61% and 12% belonged to SES II and III, respectively, while only 4% were from SES I. Only 24% resided in urban slums.

DAS scores and categories

**Figure 1:**
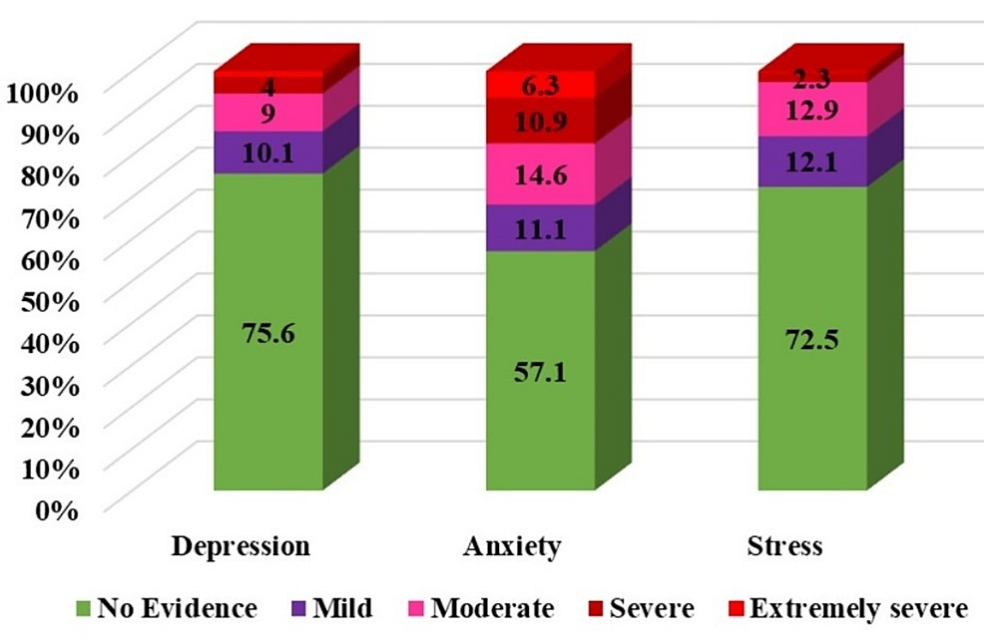
Percent distribution of categories of symptoms of depression, anxiety, and stress

The medians of depression, anxiety, and stress scores were 4 (IQR = 7), 6 (IQR = 10), and 10 (IQR = 10), respectively. Some symptoms of DAS were present in 24.4%, 42.9%, and 27.5%, respectively (Figure 1). The proportion of students with symptoms of anxiety was relatively higher.

Out of 152 study participants who had some symptoms of depression, 58.55% (89/152) had symptoms of moderate to extremely severe (SMES) depression. Similarly, out of 267 study participants who had some symptoms of anxiety, 74.16% (198/267) had SMES anxiety, and out of 171 study participants who had some symptoms of stress, 56.14% (96/171) had moderate to severe symptoms of stress.

DAS scores had strong correlations with each other as revealed by Spearman’s correlation coefficient.

Emotional intelligence and its categories

**Figure 2:**
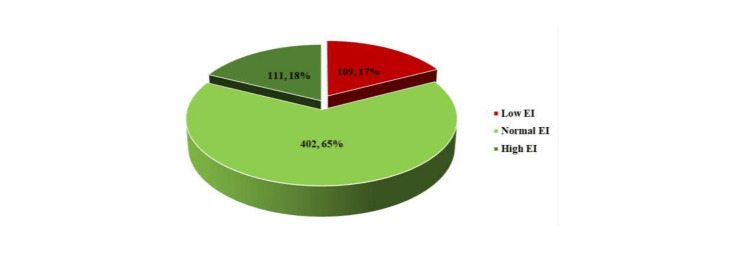
Percentage of categories of emotional intelligence (EI)

As shown in Figure 2, low EI was observed in 18% (111/622) of study participants. While EI was normal in 64.4% (402/ 622) of study participants.

The median EI score observed was 126 (IQR = 17), which was in the normal range as per conventions laid by inventors of SET. For girls, it was higher than boys but the difference was not statistically significant (125.07 vs. 125.07, p = 0.128).

Factors associated with both low EI and SMES DAS

Table 2 shows some socio-demographic factors that were associated with both low EI as well as all of the SMES DAS.

**Table 2:** Factors associated with all of SMES DAS and low EI SMES: symptoms of moderate to extremely severe; MES: moderate to extremely severe; DAS: depression, anxiety, and stress; EI: emotional intelligence.

Determinant of DAS/predictor of low EI	MES, p (OR)			Low EI
Residence in slum	<0.0001 (1.934)	<0.0001 (1.773)	0.049	0.023 (1.96)
Mother’s education less than high school certificate	<0.0001 (2.491)	<0.0001 (2.209)	0.017 (1.609)	<0.0001 (2.802)
Father’s education less than high school certificate	0.001 (1.971)	<0.0001 (1.962)	0.033 (1.521)	<0.0001 (2.189)
Co-educational schools	<0.0001 (1.997)	<0.0001 (1.549)	0.033 (1.488)	0.015 (1.514)

Qualitative analysis

**Figure 3:**
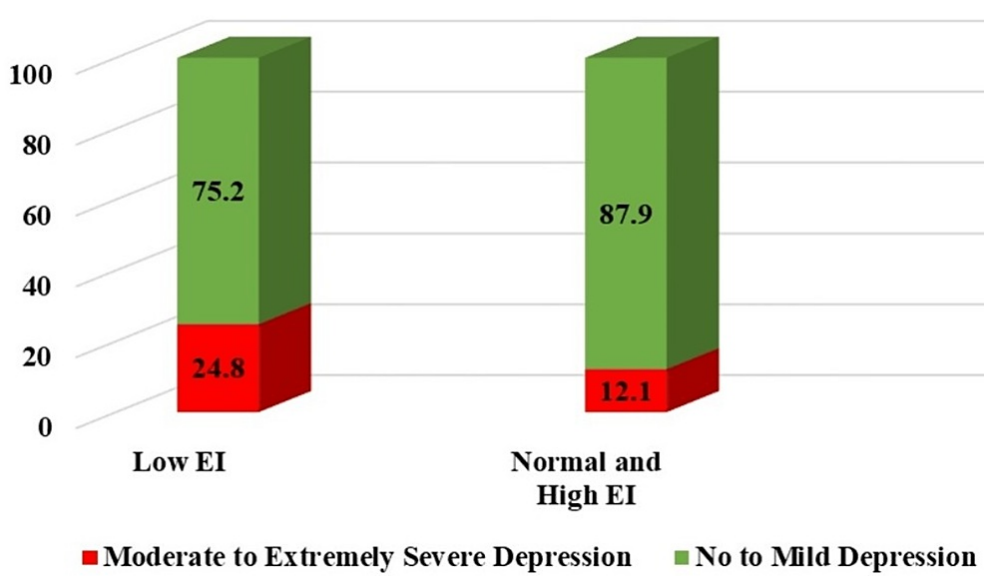
Symptoms of MES depression among EI categories MES: moderate to extremely severe; EI: emotional intelligence.

Low EI was associated with SMES depression and the identified association was highly significant (p = 0.001, OR = 2.050, 95% CI = 1.371-3.063) (Figure 3). SMES depression was present in 24.8% of participants with low EI as compared to 12.1% with normal to high EI.

**Figure 4:**
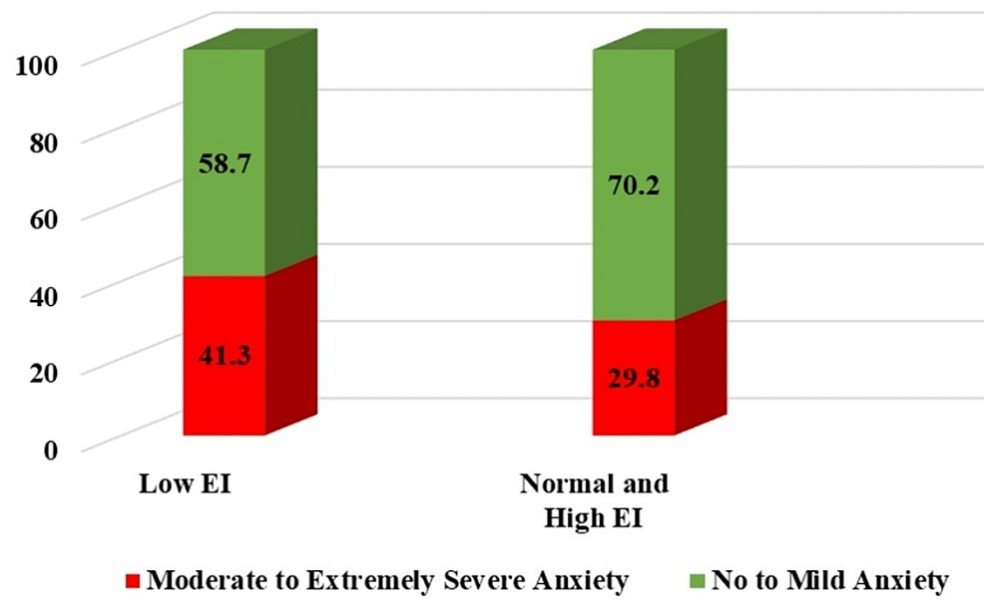
Symptoms of anxiety among EI categories EI: emotional intelligence.

Similarly, low EI was significantly associated with SMES anxiety (p = 0.02, OR = 1.384, 95% CI = 1.067-1.96) (Figure 4). SMES anxiety was present in 41.3% of study participants with low EI vs. in 29.8% of participants with normal to high EI.

**Figure 5:**
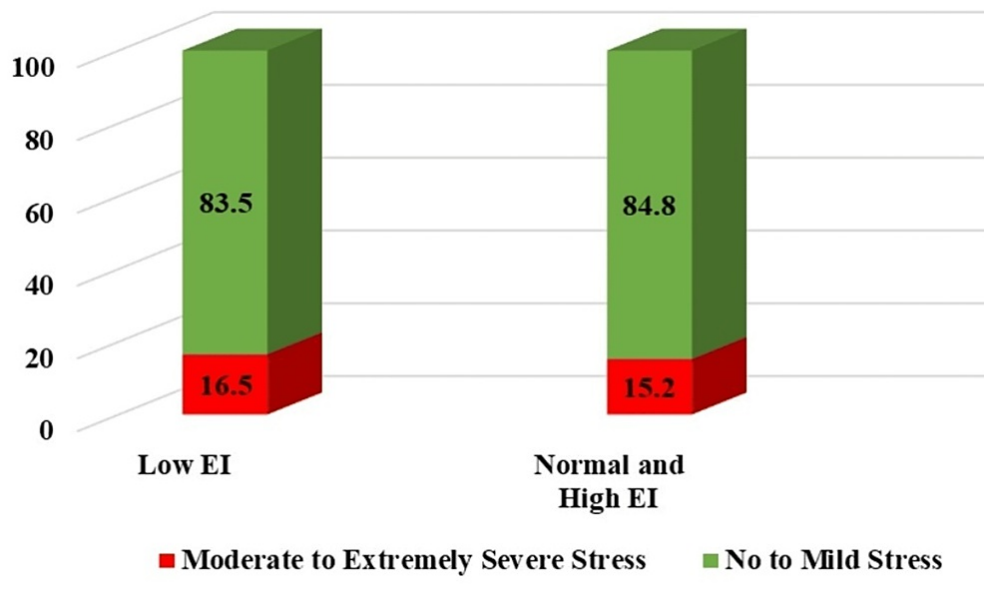
Symptoms of stress among EI categories EI: emotional intelligence.

There was no significant association identified between low EI and SMES stress (p = 0.229, OR = 0.314, 95% CI = 0.042-2.350) (Figure 5).

Quantitative analysis

As the EI scores decreased, the depression scores increased and the correlation between them was significantly negative (r = -0.221, p < 0.0001). Also, the EI score had a negative correlation with the anxiety score (r = - 0.152, p = 0.001).

The EI score of the study participants had a negative correlation with their stress scores but it was not statistically significant (r = -0.022, p = 0.611).

## Discussion

Low EI was significantly associated with the presence of symptoms of moderate to extremely severe depression and anxiety in our study participants. Similarly, there was a significant negative correlation between EI scores and depression and anxiety scores. However, low EI was not found to be significantly associated or correlated with stress.

A multitude of factors influence EI and MH of an individual since birth, as observed by us also [17,18]. But higher EI levels protect people from MHDs and people with MHDs have a significantly lower level of EI than the general population [19]. Thus, low EI is a direct psychological parameter that can be easily detected by screening utilizing available tools [20]. As pointed out in the literature, in the present study, we have presumed that the occurrence of low EI precedes SMES, if any, in most of our study participants with low EI and acts as a precursor of MH derangements, though vice versa can be true in very few study participants. Though similar factors influence low EI and the development of SMES DAS or other MH derangements (Table 2), low EI can be screened easily and well before symptoms of DAS or other MHDs occur or are ignored. It could be the advantage of such a screening program as once SMES of DAS or other MHDs occur, there is a lifetime risk of the development of MHDs [19].

Our findings are coherent with that of Shabani et al., who reported that EI explained about 35.7% of the variance in MH of their study participants who were 14 to 17 years old in Gorgan City, north of Iran [20]. Moeller et al., in their cross-sectional study in 2019 among 2094 adolescents in Middlebury, Vermont, also found that EI components as per the Trait Meta-Mood Scale (TMMS) as attention, emotional clarity, and emotional repair had a strong negative correlation with DAS [21]. Quintana-Orts et al. reported that high EI is associated with adaptive cognitive emotion regulation strategies while low EI is associated with suicidal ideation [22].

The presence of symptoms of DAS, especially of moderate to extremely severe degrees, are in turn the precursors of lifetime risk of MHDs, which are among the leading causes of illness and disability among adolescents. Such symptoms are duly reflected in the psychosocial functioning of adolescents like school work and school attendance. They may be recognized at the earliest by school teachers, staff, and peers before parents. But by that time, the severity of DAS may have increased giving rise to frank MHD. It can be followed by social stigma and withdrawal, which can exacerbate isolation, loneliness, and adverse consequences of MHDs [23].

Our study has tried to fulfill the gap in qualitative as well as quantitative assessment of the relationship between low EI as a precursor of MHDs among adolescents in the study area and India. It reiterated the role of low EI in the development of MHDs. If adolescents are periodically screened for low EI and intervened accordingly, it can help to halt the emergence of symptoms of DAS and other MH derangements, which can help to decrease the incidence of MHDs.

The generalizability of the results is restricted to school-going adolescents in the study area. Further, adolescents without access to online education were excluded from the present study and needed a different study design. Self-reported research instruments helped to eliminate reporting bias of interviews but still, it has a possible respondent bias that we tried to minimize by online education about how to prepare mind and think while completing self-reported questionnaires. There may be some social desirability bias in capturing the MH status of study subjects fully. Cross-sectional study design prevents the establishment of causation and longitudinal studies are better to find exact associations and correlations but they have ethical restrictions. Also, such studies are recommended using homogeneous samples based on gender, socio-economic status, school types, etc.

## Conclusions

We conclude that low EI among adolescents can be taken as a precursor and is a modifiable risk factor for future MHDs, especially for mood disorders such as depression and anxiety among school-going adolescents. We recommend the commencement of mechanisms to carry out periodic assessments of MH status through schools, including EI and identification of adolescents with low EI. Implementation of measures to improve their EI will prove to be more cost-effective and cost-beneficial and ultimately lead to better academic achievement, success, and satisfaction in their lives.

Integrated efforts by psychologists, counselors, and school authorities could develop EI training programs to implement in the daily school routine of adolescents and it will be the most feasible approach. Mental health policy in India at all levels, including the school level, needs a major inclusion of promotive and preventive strategies continuing curative ones.

## References

[1] Garner L (2015). Creative expression: effectiveness of a weekly craft group with women who have experienced trauma. Open J Nurs.

[2] Xiao H, Carney DM, Youn SJ, Janis RA, Castonguay LG, Hayes JA, Locke BD (2017). Are we in crisis? National mental health and treatment trends in college counseling centers. Psychol Serv.

[3] Radhakrishnan R, Andrade C (2012). Suicide: an Indian perspective. Indian J Psychiatry.

[4] Srivastava K, Chatterjee K, Bhat PS (2016). Mental health awareness: the Indian scenario. Ind Psychiatry J.

[5] Dalky HF, Gharaibeh A (2019). Depression, anxiety, and stress among college students in Jordan and their need for mental health services. Nurs Forum.

[6] Wapaño MR (2021). Emotional intelligence and mental health among adolescents. Int J Res Innov Soc Sci.

[7] Ghahramani S, Jahromi AT, Khoshsoroor D, Seifooripour R, Sepehrpoor M (2019). The relationship between emotional intelligence and happiness in medical students. Korean J Med Educ.

[8] Cotruş A, Stanciu C, Bulborea AA (2012). EQ vs. IQ which is most important in the success or failure of a student?. Procedia Soc Behav Sci.

[9] Kousha M, Bagheri HA, Heydarzadeh A (2018). Emotional intelligence and anxiety, stress, and depression in Iranian resident physicians. J Family Med Prim Care.

[10] Trigueros R, Padilla AM, Aguilar-Parra JM, Rocamora P, Morales-Gázquez MJ, López-Liria R (2020). The influence of emotional intelligence on resilience, test anxiety, academic stress and the Mediterranean diet. A study with university students. Int J Environ Res Public Health.

[11] Shaikh BM, Doke PP, Gothankar JS (2018). Depression, anxiety, stress, and stressors among rural adolescents studying in Pune and a rural block of Nanded district of Maharashtra, India. Indian J Public Health.

[12] Salguero JM, Palomera R, Fernández-Berrocal P (2012). Perceived emotional intelligence as predictor of psychological adjustment in adolescents: a 1-year prospective study. Eur J Psychol Educ.

[13] Schutte NS, Malouff JM, Bhullar N (2009). The assessing emotions scale. Assessing Emotional Intelligence. The Springer Series on Human Exceptionality.

[14] Shayan NA, Niazi A, Waseq AM, Ozcebe H (2021). Depression, Anxiety, and Stress Scales 42 (DASS-42) in Dari-language: validity and reliability study in adults, Herat, Afghanistan. Bezmialem Sci.

[15] (2023). Psychology Foundation of Australia. Depression Anxiety Stress Scales (DASS). https://www2.psy.unsw.edu.au/dass/.

[16] Ain SN, Khan ZA, Gilani MA (2021). Revised Kuppuswamy scale for 2021 based on new consumer price index and use of conversion factors. Indian J Public Health.

[17] Pepin C, Muckle G, Moisan C, Forget-Dubois N, Riva M (2018). Household overcrowding and psychological distress among Nunavik Inuit adolescents: a longitudinal study. Int J Circumpolar Health.

[18] Alavi M, Mehrinezhad SA, Amini M, Parthaman Singh MK (2017). Family functioning and trait emotional intelligence among youth. Health Psychol Open.

[19] Sánchez-Núñez MT, García-Rubio N, Fernández-Berrocal P, Latorre JM (2020). Emotional intelligence and mental health in the family: the influence of emotional intelligence perceived by parents and children. Int J Environ Res Public Health.

[20] Shabani J, Damavandi AJ (2011). The importance of gender as a moderator for the relationship between emotional intelligence and mental health of adolescents. Asian Soc Sci.

[21] Moeller RW, Seehuus M, Peisch V (2020). Emotional intelligence, belongingness, and mental health in college students. Front Psychol.

[22] Quintana-Orts C, Mérida-López S, Rey L, Neto F, Extremera N (2020). Untangling the emotional intelligence-suicidal ideation connection: the role of cognitive emotion regulation strategies in adolescents. J Clin Med.

[23] Konjengbam E, Bigyabati R, Suriya P, Rajkumari B (2023). Mental health issues among adolescents in Manipur: a cross sectional study. Int J Acad Med Pharm.

